# HIV-related risk among justice-involved young African American women in the U.S. South

**DOI:** 10.1186/s40352-023-00228-7

**Published:** 2023-08-24

**Authors:** Felicia A. Browne, Yukiko Washio, William A. Zule, Wendee M. Wechsberg

**Affiliations:** 1 Substance Use, Gender, and Applied Research Program, RTI International, 3040 East Cornwallis Road, NC 27709-2194 Research Triangle Park, USA; 2 Health Behavior, University of North Carolina Gillings School of Global Public Health, 135 Dauer Drive, Chapel Hill, NC 27599 USA; 3 Department of Obstetrics, Gynecology and Reproductive Sciences, Temple University Lewis Katz School of Medicine, Philadelphia, PA 19140 USA; 4 Department of Psychology, North Carolina State University, 640 Poe Hall, Campus, Box 7650, Raleigh, NC 27695 USA; 5 Psychiatry and Behavioral Sciences, Duke University School of Medicine, 40 Duke Medicine Circle, Durham, NC 27710 USA; 6 Maternal and Child Health, University of North Carolina Gillings School of Global Public Health, 135 Dauer Drive, Chapel Hill, NC 27599 USA

**Keywords:** Arrest, Justice involvement, Young African American women, Emerging adulthood, HIV, Substance use, Interventions, Service needs

## Abstract

Incarceration rates have increased exponentially among women, and racial disparities in justice involvement persist. Coupled with disparities in HIV in the US South that begin early in the life course, it is important to explicate the relationship between justice involvement, HIV-related risk (such as illicit drug use and sexual risk), and service needs for young African American women. This study examined the association of previous arrest, biological and self-reported HIV-related risk, and reported service needs at baseline among 646 African American women aged 18 to 25 who were recruited as part of an HIV-risk reduction trial. Approximately 24% of participants reported previously being arrested. In adjusted analyses, several substance use and sexual risk variables were found to be significant, including increased odds of positive screens for both cocaine (AOR: 3.09; 95% CI [1.49, 6.41]) and marijuana (AOR: 1.82; 95% CI [1.17, 2.83]), trading sex for goods (AOR: 2.23; 95% CI [1.14, 4.38]), and recent sexually transmitted infections (AOR: 1.84; 95% CI [1.03, 3.27]). Previous arrest was associated with greater service needs, including violence-related (AOR: 4.42; 95% CI [2.03, 9.64]), parenting (AOR: 2.92; 95 CI% [1.65, 5.17]), and housing (AOR: 2.38; 95% CI [1.54, 3.67)]). The study findings indicate the increased risk across both HIV-related substance use and sexual risk and the service needs for African American women in emerging adulthood who have been arrested. These disparities suggest the importance of interventions to address the specific needs of this population at a critical period to not only prevent HIV but also address social determinants.

## Introduction

The number of women incarcerated—defined as being in jail, prison, or other confined location overnight—in the United States rose 700% between 1980 (*n* = 26,378) and 2019 (*n* = 222,455) (The Sentencing Project, 2020; U.S. Department of Justice, 2021). In 2018–2019, an additional one million women were on probation (*n* = 885,000) or parole (*n* = 114,140) (The Sentencing Project, 2020). A report that compiled data on incarceration among women concluded that 231,000 women were incarcerated in 2019 in the United States, and that incarceration rates among women were increasing twice as fast as among men (Kajstura, 2019). These increases appear to be driven largely by harsher drug sentencing laws and barriers to reentry that disproportionately affect women (The Sentencing Project, 2020). These barriers include providing for their children and families, as they are often the primary caregiver, and securing employment and stable housing (Substance Abuse & Mental Health Services Administration, 2020).

In addition to the consideration of gender, there are marked differences in justice involvement by race. Involvement in the criminal justice system disproportionately affects African Americans (The Sentencing Project, 2020). In 2020, African American residents had the highest imprisonment rate of any race or ethnic group in the U.S. (938 per 100,000), ranging from 1.2 to 12.7 times greater than other racial or ethnic groups (Carson, 2021). Both African American men and women have higher rates of incarceration than their White counterparts, with African American men having 5.7 times the rate of White men and African American women having 2.3 times the rate of White women (Carson, 2020). Among girls 10 to 17 years of age, incarceration rates are over three times higher among African American girls than among their non-Hispanic White counterparts (94 per 100,000 as compared with 29 per 100,000, respectively) (The Sentencing Project, 2020).

Another persisting disparity by race is HIV, which continues to disproportionately affect African American women compared with women from other racial/ethnic groups. Despite representing an estimated 13% of the female population, African American female adolescents and adult women represented an estimated 55% of the new diagnoses of HIV in 2019 (Centers for Disease Control & Prevention, 2021a). During this same period, the rate of HIV diagnoses for African American women was more than 12 times the rate of White women (21.3 as compared with 1.7 per 100,000).

HIV-related risks, such as illicit drug use, are often associated with justice involvement. The prevalence of illicit drug use is high—ranging from 30% to 69% internationally—among arrestees and formerly incarcerated persons (Fazel et al., 2017; Noska et al., 2016; Rowell-Cunsolo et al., 2018; Western & Simes, 2019). Previous research among women with histories of arrest or incarceration reported high prevalence of a plethora of health and social issues, including illicit drug use, homelessness, unemployment, low educational attainment, and psychological distress (Kim & Peterson, 2020; Nowotny et al., 2019; Prison Health Services Fail to Meet the Needs of Incarcerated Women, 2021; Salem et al., 2020; Swavola et al., 2016). Many women who have been incarcerated also have histories of physical and sexual victimization (Browne et al., 1999; Fogel & Belyea, 1999; Jones et al., 2018; Ravi et al., 2007). Studies have shown that histories of childhood sexual abuse and other types of childhood trauma are linked to substance use (Kittirattanapaiboon et al., 2017; Pergolizzi et al., 2012) and sexual risk among adolescents and psychological distress and substance use disorders among adult women (Draucker & Mazurczyk, 2013; Kittirattanapaiboon et al., 2017; Pergolizzi et al., 2012; Prangnell et al., 2020; Schäfer & Fisher, 2011).

Alcohol and other drug use, mental health problems, unemployment, housing instability, and violence are common among women involved in the justice system. Among women in jail, 82% have experienced alcohol or other drug abuse or dependence in their lifetime; 32% have experienced serious mental illness, such as major depression, bipolar disorder, or schizophrenia; and 60% were unemployed at the time of arrest (Swavola et al., 2016). In a prebooking diversion program in King County, Washington, 80% of women were experiencing homelessness. A recent review article found high rates of childhood sexual abuse (50%–66%), adult sexual assault (28%–68%), and lifetime sexual assault (56%–82%) among women who were incarcerated (Karlsson & Zielinski, 2020).

Some longitudinal studies have tried to examine the directionality of the relationship between justice involvement and HIV risk. In a multicity longitudinal cohort study of women at risk for HIV over 17 years (median follow-up period per woman of 5.5 years), almost half the cohort reported being incarcerated during or before the study (Knittel et al., 2021). Based on these high levels of incarceration, the authors concluded that women who have experienced incarceration are a key population for HIV prevention interventions. Another study reported on data from over 5,000 women, 75% of whom were African American, who participated in the 2013 National HIV Behavioral Surveillance Survey that was conducted in 20 U.S. cities. In that study, 11% of women reported being incarcerated in the past 12 months and another 36% reported ever being incarcerated (Wise et al., 2017). Incarceration was associated with higher prevalence of HIV risk behaviors, including condomless sex with a higher number of casual partners, exchanging sex for money or drugs, injection drug use, noninjection drug use, and higher prevalence of HIV (Wise et al., 2017).

These high levels of HIV risk behaviors, persisting racial disparities in HIV and justice involvement, and unmet service needs highlight the need for interventions that address intersecting issues among African American women who are involved in the justice system. Moreover, the high rates of incarceration among African American adolescent girls indicate the importance of reaching young women at this critical period of emerging adulthood.

One potentially important unanswered question is how engagement in the justice system—that is, being arrested—is associated with different indicators of HIV risk within a key population of young adults at risk for HIV. Specifically elucidating which indicators of risk and service needs are significant, after accounting for socio-demographic characteristics, may provide important evidence for prioritizing interventions and programs. In this article, quantitative baseline data are analyzed and presented from a sample of young African American women who were currently using alcohol or other drugs and reported sexual risk. Among this group of young women, we examine differences between women with a history of arrest and women without a history of arrest.

## Methods

This research used baseline data from the North Carolina Young Women’s CoOp, a National Institutes of Health-funded R01 trial based in health departments. This study enrolled 652 African American women aged 18 to 25 who reported recent condomless sex and substance use (alcohol and/or other drugs) (Browne et al., 2018). Conducted in three North Carolina counties, this trial tested the delivery of an HIV risk-reduction intervention. Participants were recruited primarily through targeted in-person street outreach, clinic in-reach and participant referrals. At study enrollment, participants completed an audio-computer assisted self-interview (ACASI) administered risk behavior assessment (Wechsberg, 1998), alcohol testing via breathalyzer, and urine drug screening for 10 drugs. At the end of this appointment, they were referred to the health department for HIV, gonorrhea, and chlamydia testing. Additional details about the baseline and trial study procedures are available via the published protocol (Browne et al., 2018).

## Measures

### Outcomes

Several substance use and sexual risk factors related to HIV were assessed via the risk behavior instrument and through biological testing and medical record retrieval for sexually transmitted infection (STI) testing. Substance use variables included self-reported heavy alcohol use in the past month (having four or more drinks on one occasion), a participant’s or their partner’s use of alcohol or other drugs at last sex, and positive urine drug screens for marijuana and cocaine. Sexual risk variables included last sex without a condom, ever traded sex (had sex for drugs, money, food, clothing, a place to stay or any other goods), having more than one partner in the past 3 months, and positive STI test (biologically confirmed at enrollment, in addition to self-reported lifetime and within the past 6 months). Service needs were assessed through several dichotomous questions asking whether participants thought they needed certain services. These services included violence-related, health care, housing, education, alcohol and other drug treatment, parenting, and any other services (that the participant specified). The specified responses to the other service question were reviewed; if the response was related to one of the other six services, they were subsequently recoded—for example, GED (recoded to education) and childcare (recoded to parenting).

### Exposure

Previous arrest was assessed by a dichotomous question that asked whether participants had ever been arrested.

### Covariates

Several demographic variables were included given the importance of these factors in both justice involvement and HIV-risk behavior. Age (recoded into two categories—18–20 and 21–25), whether a participant had a main partner in the past 3 months (dichotomous), whether a participant had children (binary), education (both attainment and enrollment status), employment status (dichotomous), housing instability (a dichotomous question asking whether a participant was currently experiencing homelessness), and food insecurity (any instance of people in household going without food; recoded from a question asking how often people in a participant’s home are without food, ranging from Never to Every Week, resulting in a binary variable).

### Analysis

We conducted a complete case analysis because very few data on the selected variables were missing (< 1%), resulting in an analytic sample of 646, with the exception of the biological STI variable, which was based on retrieval of medical records (*N* = 600). Chi-square tests were conducted to examine associations between previous incarceration status and demographics. To adjust for potential confounding, all demographic variables were retained for the logistic regression models for substance use, sexual risk, and service needs. Analyses were conducted in Stata MP/IC 16.1.

## Results

Table 1 presents the participant characteristics overall and by incarceration status. Approximately 24% of the participants reported ever being arrested. Among participants who had been previously arrested, the mean number of arrests was 2.7 and the median was 2 (not shown). Participants who had been arrested were older, more likely to have a main partner, less likely to have completed some college or higher level of education, less likely to be currently in school, more likely to be currently unstably housed, and more likely to have experienced food insecurity. The only variable not statistically significant was employment status.

**Table 1:** Sample sociodemographic characteristics at baseline, overall, and by previous arrest (*N* = 646)

	**Total**		**Never Arrested**		**Arrested**			
**Variable**	**(*N* = 646)**		**(*N* = 492)**		**(*N* = 154)**			
	**N**	**%**	**N**	**%**	**N**	**%**	$\chi^2$	***p*-value**
Age							43.65*	< 0.001
18–20	292	(45.2)	258	(52.4)	34	(22.1)		
21–25	354	(54.8)	234	(47.6)	120	(77.9)		
Main partner, past 3 months	511	(79.1)	374	(76.0)	137	(89.0)	11.89*	0.001
Have child(ren)	234	(36.2)	138	(28.0)	96	(62.3)	59.69*	< 0.001
Completed some college or higher	334	(51.7)	272	(55.3)	62	(40.3)	10.60*	0.001
Currently in school	330	(51.1)	282	(57.3)	48	(31.2)	32.09*	< 0.001
Employed	375	(58.0)	288	(58.5)	87	(56.5)	0.20	0.654
Currently unstably housed	86	(13.3)	44	(8.9)	42	(27.3)	34.15*	< 0.001
Food insecurity	166	(25.7)	117	(23.8)	49	(31.8)	3.97*	0.046

^*^p < .05

Table 2 presents the unadjusted and adjusted models of HIV-related substance use and sexual risk. Adjusting for the sociodemographic variables, women who had been arrested previously had 1.82 times the odds of having a positive marijuana screen at baseline, more than 3 times the odds of a cocaine positive screen and 1.55 times the odds of heavy alcohol use, compared with women without a previous arrest. For sexual risk, several variables were statistically significant in adjusted models. Compared with women who had not been arrested, women who had been arrested had 1.59 times the odds of either they or their partner being impaired by alcohol or other drugs at last sex, 2.23 times the odds of ever trading sex, 1.54 times the odds of a positive STI test in their lifetime, and 1.84 times the odds of a positive STI test in the past 6 months.

**Table 2:** Unadjusted and adjusted^a^ models of HIV-related substance use and sexual risk – previous arrest (*N* = 646)

	**Total**		**Never Arrested**		**Arrested**							
**Variable**	**(*N* = 646)**		**(*N* = 492)**		**(*N* = 154)**		**OR**	**95% CI**	***p*-value**	AOR*	**95% CI**	***p*-value**
**Substance Use**												
Marijuana, Positive Screen	428	(66.3)	311	(63.2)	117	(76.0)	1.84*	(1.22—2.78)	0.004	1.82*	(1.17—2.83)	0.008
Cocaine, Positive Screen	37	(5.7)	16	(3.3)	21	(13.6)	4.70*	(2.38—9.26)	< 0.001	3.09*	(1.49—6.41)	0.002
Heavy Alcohol Use	349	(54.0)	253	(51.4)	96	(62.3)	1.56*	(1.08—2.27)	0.018	1.55*	(1.03—2.32)	0.034
**Sexual Risk**												
Condomless Last Sex	428	(66.3)	317	(64.4)	111	(72.1)	1.43	(0.96—2.12)	0.081	1.02	(0.66—1.57)	0.946
Impaired Last Sex, Self or Partner	305	(47.2)	216	(43.9)	89	(57.8)	1.75*	(1.21—2.52)	0.003	1.59*	(1.07—2.37)	0.022
More Than One Partner	252	(39.0)	187	(38.0)	65	(42.2)	1.19	(0.82—1.72)	0.351	1.50	(1.00—2.25)	0.051
Ever Traded Sex	47	(7.3)	25	(5.1)	22	(14.3)	3.11*	(1.70—5.70)	< 0.001	2.23*	(1.14—4.38)	0.019
Positive STI Test (Lifetime)	208	(32.2)	145	(29.5)	63	(40.9)	1.66*	(1.14—2.41)	0.008	1.54*	(1.02—2.30)	0.038
Positive STI Test, Past 6 Months	77	(11.9)	53	(10.8)	24	(15.6)	1.53	(0.91—2.57)	0.110	1.84*	(1.03—3.27)	0.039
Positive STI Test (Biologically confirmed) (*N* = 600)	100	(16.7)	76	(16.5)	24	(17.1)	1.05	(0.63—1.73)	0.863	1.27	(0.73—2.22)	0.400

*OR/AOR* Odds ratio/adjusted odds ratio, *CI* Confidence interval, *STI* Sexually transmitted infection

^*^p < .05

^a^Variables adjusted for age, main partner, having children, education (attainment and status), employment, housing instability, and food insecurity

Unadjusted and adjusted models of service needs and previous arrest are presented in Table 3. In unadjusted models, previous arrest history was associated with increased odds of reporting the need for all six listed services. In the adjusted models, women who were previously arrested had increased odds of reporting the need for five of the six listed services, including 4.42 times the odds for services related to violence, 2.92 times the odds for parenting services, and 2.38 times the odds for housing services. The only service that was not statistically significant in either adjusted or unadjusted models was the any other service variable where participants could specify other services. The most common other services specified for this variable included employment (*N* = 13), transportation (*N* = 7), and food (*N* = 5).

**Table 3:** Unadjusted and adjusted^a^ models of service needs – previous arrest (*N* = 646)

	**Total**		**Never Arrested**		**Arrested**							
**Variable**	**(*N* = 646)**		**(*N* = 492)**		**(*N* = 154)**		**OR**	**95% CI**	***p*-value**	AOR*	**95% CI**	***p*-value**
**Need for Services**												
Violence	35	(5.4)	16	(3.3)	19	(12.3)	4.19*	(2.10—8.36)	< 0.001	4.42*	(2.03—9.64)	< 0.001
Health care	153	(23.7)	102	(20.7)	51	(33.1)	1.89*	(1.27—2.82)	0.002	1.66*	(1.06—2.58)	0.025
Housing	176	(27.2)	98	(19.9)	78	(50.6)	4.13*	(2.81—6.07)	< 0.001	2.38*	(1.54—3.67)	< 0.001
Education	187	(28.9)	116	(23.6)	71	(46.1)	2.77*	(1.90—4.05)	< 0.001	1.90*	(1.25—2.86)	0.002
Alcohol and other drug treatment	48	(7.4)	28	(5.7)	20	(13.0)	2.47*	(1.35—4.53)	0.003	1.96	(1.00—3.83)	0.050
Parenting	71	(11.0)	31	(6.3)	40	(26.0)	5.22*	(3.13—8.71)	< 0.001	2.92*	(1.65—5.17)	< 0.001
Other	31	(4.8)	24	(4.9)	7	(4.5)	0.93	(0.39—2.20)	0.866	0.60	(0.24—1.51)	0.280

*OR/AOR* Odds ratio/adjusted odds ratio, *CI* Confidence interval

^*^p < .05

^a^Variables adjusted for age, main partner, having children, education (attainment and status), employment, housing instability, and food insecurity

In sensitivity analyses of justice involvement (not shown), women who spent more than a day in jail, detention, or juvenile detention (a potential proxy for severity) were compared with women who spent less than a day. Women who spent more than one day being detained were significantly older, more likely to experience food insecurity, more likely to have ever engaged in sex trading, and more likely to need housing services and parenting services. These two groups did not differ significantly for any of the other variables assessed.

## Discussion

Approximately one of every four participants in this sample of young African American women who reported recent substance use and sexual risk behavior had been arrested previously. This finding is a concern given they are at the beginning of adulthood and how arrest history affects subsequent opportunities such as employment and education (Sweeten, 2006). This potential consequence was supported by the study findings, which showed that women with previous arrests were less likely to be in school or have attained a certain level of education and were more likely to be food insecure and unstably housed. They also were more likely to report the need for educational services. While we did not observe any differences for employment, we did not assess the type of employment, as there could be differences in the types of employment opportunities available to women who have been previously arrested, including lower hourly wages. Even after accounting for social determinants and age, previous arrest history was independently associated with greater service needs for five of the six services assessed—housing, health care, education, parenting, and violence-related. The increased odds of both HIV-related substance use and sexual risk—including STI risk and sex trading—further supports the argument for a tailored and targeted approach for young women who have been arrested. While it is possible that for some of the risks identified, such as sex trading and illicit substance use, the risks could be the actual reason for their arrest, other identified risks, such as STIs, impaired sex and heavy alcohol use are not likely to be the cause of their arrest.

Given the increased risk, HIV prevention tools such as pre-exposure prophylaxis (PrEP) to avert HIV are essential (Dauria et al., 2021), as HIV incidence among African American women in the United States has persisted (Centers for Disease Control & Prevention, 2021a). This is particularly critical as African Americans have lower rates of PrEP engagement and coverage than other populations (Centers for Disease Control & Prevention, 2021b). Additionally, social determinants and service needs are important aspects to be considered when developing HIV risk-reduction interventions and other programs for this key population. At the individual level, providing educational opportunities and job skills training to support future employability of young women with arrest histories is essential, along with substance treatment programs that address their previous experiences, including trauma. Awareness of why they are using substances is often an important step in treatment and reducing risk behavior. Additionally, it is vital that programs are implemented at the structural level, including policies that explicitly focus on businesses, educational institutions, and service organizations to address systemic barriers for people with arrest histories (Substance Abuse & Mental Health Services Administration, 2020). An important component to any effort is addressing the developmental, social, and cultural experiences of young African American women with arrest histories, so that these programs resonate with them and have the potential for the greatest impact. To ensure their experiences and voices are integrated in programs, it is critical to have their engagement and input in the development and implementation of programs through participatory activities, such as expert panels and advisory boards.

This study has some limitations regarding assessing temporality of the relationships due to the use of cross-sectional data and based on the questions asked. Unfortunately, only a few questions were included in the interview to assess their experience with the justice system, including whether they were arrested, the number of times they were arrested, and how long they spent time in jail, detention, or juvenile detention. These various types of justice involvement were included in one question, and notably, experiences can differ depending on the type of justice involvement. As a way of addressing potential severity, we did conduct sensitivity analyses to examine how those with justice involvement differed depending on the amount of time they spent arrested.

Additional studies are needed to further understand these associations, when they begin and their longitudinal impact. Age plays a key role in both risk behavior and justice involvement. Understanding whether there is a direct causal relationship between justice involvement and HIV-related risks, or whether there is another contributing factor that impacts them both will be essential and may have implications for policies and programs to intervene early in the life course for young African American women to prevent both arrest experiences and HIV-related risks during the critical period of development and transition from adolescence to adulthood.

Despite these limitations, this study did elucidate important associations that suggest areas for intervention for this key group of emerging adults. Moreover, with the current focus on justice involvement—particularly as it affects African American populations—and the acknowledgement of the importance of intersectionality, which recognizes individuals’ multiple identities and experiences and how they are shaped by them (Crenshaw, 2017) and equity (Gueta, 2020), interventions to prevent justice involvement that address important intersections related to social determinants and HIV may be well suited for community, government, and others’ buy-in. For example, interventions for women with recent justice involvement that includes HIV-risk reduction and referrals to services, is one way that has shown promising outcomes for other populations of women (Johnson et al., 2015). To ultimately have an impact and eliminate persisting disparities in health and well-being, an intersectional approach that addresses the diverse needs of young African American women who use substances will be vital.

## Data Availability

The datasets during and/or analyzed during the current study are available from the corresponding author on reasonable request.

## Abbreviations

AOR: Adjusted odds ratio
CI: Confidence interval
GED: General educational development
OR: Odds ratio
PrEP: Pre-exposure prophylaxis
STI: Sexually transmitted infection
